# Operating in a Reverberating Regime Enables Rapid Tuning of Network States to Task Requirements

**DOI:** 10.3389/fnsys.2018.00055

**Published:** 2018-11-06

**Authors:** Jens Wilting, Jonas Dehning, Joao Pinheiro Neto, Lucas Rudelt, Michael Wibral, Johannes Zierenberg, Viola Priesemann

**Affiliations:** ^1^Max-Planck-Institute for Dynamics and Self-Organization, Göttingen, Germany; ^2^Magnetoencephalography Unit, Brain Imaging Center, Johann-Wolfgang-Goethe University, Frankfurt, Germany; ^3^Bernstein-Center for Computational Neuroscience, Göttingen, Germany

**Keywords:** adaptation, collective dynamics, neural network, cognitive states, neuromodulation, criticality, balanced state, hierarchy

## Abstract

Neural circuits are able to perform computations under very diverse conditions and requirements. The required computations impose clear constraints on their fine-tuning: a rapid and maximally informative response to stimuli in general requires decorrelated baseline neural activity. Such network dynamics is known as asynchronous-irregular. In contrast, spatio-temporal integration of information requires maintenance and transfer of stimulus information over extended time periods. This can be realized at criticality, a phase transition where correlations, sensitivity and integration time diverge. Being able to flexibly switch, or even combine the above properties in a task-dependent manner would present a clear functional advantage. We propose that cortex operates in a “reverberating regime” because it is particularly favorable for ready adaptation of computational properties to context and task. This reverberating regime enables cortical networks to interpolate between the asynchronous-irregular and the critical state by small changes in effective synaptic strength or excitation-inhibition ratio. These changes directly adapt computational properties, including sensitivity, amplification, integration time and correlation length within the local network. We review recent converging evidence that cortex *in vivo* operates in the reverberating regime, and that various cortical areas have adapted their integration times to processing requirements. In addition, we propose that neuromodulation enables a fine-tuning of the network, so that local circuits can either decorrelate or integrate, and quench or maintain their input depending on task. We argue that this task-dependent tuning, which we call “dynamic adaptive computation,” presents a central organization principle of cortical networks and discuss first experimental evidence.

Cortical networks are confronted with ever-changing conditions, whether these are imposed on them by a natural environment, or induced by the actions of the subjects themselves. For example, when a predator is lurking for a prey it should detect the smallest movement in the bushes anywhere in the visual field, but as soon as the prey is in full view and the predator moves to strike, visual attention should focus on the prey (Figure 1C). Optimal adaptation for these changing tasks requires a precise and flexible adjustment of input amplification and other properties within the local, specialized circuits of primary visual cortex: strong amplification of small input while lurking, but quenching of any irrelevant input when chasing. These are changes from one task to another. However, even the processing within a single task may require the joint contributions of networks with diverse computational properties. For example, listening to spoken language involves the integration of phonemes at the timescale of milliseconds to words and whole sentences lasting for seconds. Such temporal integration might be realized by a hierarchy of temporal receptive fields, a prime example of adaption to different processing requirements of each brain area (Murray et al., 2014; Hasson et al., 2015, Figure 1B).

**Figure 1:**
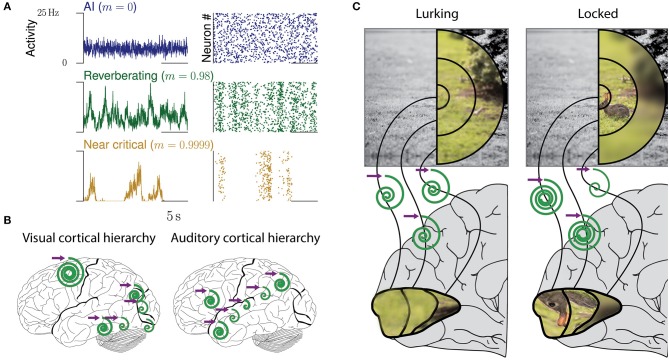
Collective dynamics of cortical networks. **(A)** Examples of collective spiking dynamics representing either irregular and uncorrelated activity (blue), reverberations (green), or dynamics close to a critical state (yellow). Population spiking activity and raster plots of 50 neurons are shown. **(B)** Hierarchical organization of collective cortical dynamics. In primary sensory areas, input is maintained and integrated only for tens of milliseconds, whereas higher areas show longer reverberations and integration. The purple arrow represents any input to the respective area, the spirals the maintenance of the input over time (inspired from Hasson et al., 2015). **(C)** Dynamic adaptation of collective dynamics in local circuits. When a predator is lurking for prey, the whole field of view needs to be presented equally in cortex. Upon locking on prey, attention focuses on the prey. This could be realized by local adaptation of the network dynamics, which amplifies the inputs from the receptive fields representing the rabbit (“tuning in”), while quenching others (“tuning out”).

Basic network properties like sensitivity, amplification, and integration timescale optimize different aspects of computation, and hence a generic input-output relation can be used to infer signatures of the computational properties, and changes thereof (Kubo, 1957; Wilting and Priesemann, 2018a). Throughout this manuscript, we refer to *computation* capability in the following two, high-level senses. First, the integration timescale determines the capability to process sequential stimuli. If small inputs are quenched away rapidly, the network may quickly be ready to process the next input. In contrast, networks that maintain input for long timescales may be slow at responding to novel input, but instead they can integrate information and input over extended time periods (Bertschinger and Natschläger, 2004; Lazar, 2009; Boedecker et al., 2012; Del Papa et al., 2017). This is at the heart of reservoir computing in echo state networks or liquid state machines (Buonomano and Merzenich, 1995; Maass et al., 2002; Jaeger and Haas, 2004; Schiller and Steil, 2005; Jaeger et al., 2007; Boedecker et al., 2012). Second, the detection of small stimuli relies on a sufficient amplification (Douglas et al., 1995). However, increased sensitivity to weak stimuli can lead to increased trial-to-trial variability (Gollo, 2017).

These examples show that local networks that are tuned to one task may perform worse at a different one, and there is no one-type-fits-all network for every environmental and computational demand. How does a neural network manage to both react quickly to new inputs when needed, but also maintain memory of the recent input, e.g., when a human listens to language? Did the brain evolve a large set of specialized circuits, or did it develop a manner to fine-tune its circuits quickly to the computational needs? A flexible tuning of response properties would be desirable in the light of resource and space constraints. Indeed, in experiments one of the most prominent features of cortical responses is their strong dependence on cognitive state and context. For example, the cognitive state clearly impacts the strength, delay and duration of responses, the trial-to-trial variability, the network synchrony, and the cross-correlation between units (Kisley and Gerstein, 1999; Massimini et al., 2005; Poulet and Petersen, 2008; Curto et al., 2009; Goard and Dan, 2009; Kohn et al., 2009; Harris and Thiele, 2011; Marguet and Harris, 2011; Priesemann et al., 2013; Scholvinck et al., 2015). Transitions between different cognitive states have been described by phase transitions (Galka et al., 2010; Steyn-Ross and Steyn-Ross, 2010; Steyn-Ross et al., 2010).

While a phase transition can be very useful to realize cognitive state changes, we here want to emphasize a particular property of systems close to phase transitions: without actually crossing the critical point, already small parameter changes can have a large impact on the network dynamics and function. Hence a classical phase transition may not be necessary for adaptation. In addition to the well-established phase transitions, adaptation could be realized as a dynamic process that regulates the proximity to a phase transition and allows cortical networks to fine-tune their sensitivity, amplification, and integration timescale within one cognitive state, depending on the specific requirements. In order to allow efficient adaptation, cortical networks must evidently satisfy the following requirements. (i) The network properties are easily tunable to changing requirements, e.g., the required synaptic or neural changes should be small. (ii) The network is fully functional in its ground state, and also in the entire vicinity, i.e., the adaptive tuning does not destabilize or dysfunctionalize it. (iii) The network receives, modifies and transfers information according to its needs, e.g., it amplifies or quenches the input depending on task. (iv) The network's ground state in general should enable integration of input over any specific past window, as required by a given task.

We propose that cortex operates in a particular dynamic regime, the “reverberating regime”, because in this regime small changes in neural efficacy can tune computational properties over a wide range – a mechanism that we propose to call *dynamic adaptive computation*. In this regime a cortical circuit can interpolate between two states described below, which both have been hypothesized to govern cortical dynamics and optimize different aspects of computation (Burns and Webb, 1976; Softky and Koch, 1993; Vreeswijk and Sompolinsky, 1996; Brunel, 2000; Beggs and Plenz, 2003; Stein et al., 2005; Beggs and Timme, 2012; Plenz and Niebur, 2014; Tkačik et al., 2015; Humplik and Tkačik, 2017; Muñoz, 2017; Wilting and Priesemann, 2018a,b). In the following, we recapitulate the computational properties of these two states and then identify recent converging evidence that in fact the reverberating regime governs cortical dynamics *in vivo*. We then show how specifically the reverberating regime can combine the computational properties of the two extreme states while maintaining stability and thereby satisfies all requirements for cortical network function postulated above. Finally, we outline future theoretical challenges and experimental predictions.

One hypothesis suggests that spiking statistics in the cortical ground state is asynchronous and irregular (Burns and Webb, 1976; Softky and Koch, 1993; Stein et al., 2005), i.e., neurons spike independently of each other and in a Poisson manner (Figure 1A). Such dynamics may be generated by a “balanced state,” which is characterized by weak recurrent excitation compared to inhibition (Vreeswijk and Sompolinsky, 1996; Brunel, 2000). The typical balanced state minimizes redundancy, has maximal entropy in its spike patterns, and supports fast network responses (Vreeswijk and Sompolinsky, 1996; Denève and Machens, 2016). The other hypothesis proposes that neuronal networks operate at criticality (Bienenstock and Lehmann, 1998; Beggs and Plenz, 2003; Levina et al., 2007; Beggs and Timme, 2012; Plenz and Niebur, 2014; Tkačik et al., 2015; Humplik and Tkačik, 2017; Muñoz, 2017; Kossio et al., 2018), and thus in a particularly sensitive state at a phase transition. This state is characterized by long-range correlations in space and time, and in models optimizes performance in tasks that profit from extended reverberations of input in the network (Bertschinger and Natschläger, 2004; Haldeman and Beggs, 2005; Kinouchi and Copelli, 2006; Wang et al., 2011; Boedecker et al., 2012; Shew and Plenz, 2013; Del Papa et al., 2017). These two hypotheses, asynchronous-irregular and critical, can be interpreted as the two extreme points on a continuous spectrum of response properties to minimal perturbations, the first quenching any rate perturbation quickly within milliseconds, the other maintaining it for much longer. Hence the two hypotheses clearly differ already in the basic response properties they imply.

A general first approach to characterize the response properties of any dynamical system is based on linear response theory: When applying a minimal perturbation or stimulation, e.g., adding a single extra spike to neuron *i*, the basic response is characterized by *m*_*i*_, the number of *additional* spikes triggered in all postsynaptic neurons (London et al., 2010), which can be interpreted as efficacy of the one neuron. If the efficacy is sufficiently homogeneous across neurons, then the average *neural efficacy* *m* represents a control parameter, and quantifies the impact of any single extra spike in a neuron on its postsynaptic neurons, and thus the basic network response properties to small input. In the next step, any of these triggered spikes in turn can trigger spikes in a similar manner, and thereby the small stimulation may cascade through the network. The network response may vary from trial to trial and from neuron to neuron, depending on excitation-inhibition ratio, synaptic strength, and membrane potential of the postsynaptic neurons. Thus *m* does not describe each single response, but the expected (average) response of the network, and thereby enables an assessment of the network's stability and computational properties. The magnitude of the neural efficacy *m* defines two different response regimes: If one spike triggers on average less than one spike in the next time step (*m* < 1), then any stimulation will die out in finite time. For *m* > 1, stimuli can be amplified infinitely, and *m* = 1 marks precisely the transition between stable and unstable dynamics (Figure 2B). In addition, *m* directly determines the amplification of the stimulus, the duration of the response, the intrinsic network timescale and the response variability, among others in the framework of autoregressive processes (Harris, 1963; Wilting and Priesemann, 2018a,b). Although details may depend on the specific process or model, many results presented in the following are qualitatively universal across diverse models that show a (phase) transition from stable to unstable, from ordered to chaotic, or from non-oscillatory to oscillatory activity. These include for example AR(1), Kesten, branching, and Ornstein-Uhlenbeck processes, systems that show a Hopf bifurcation, or systems at the transition to chaos (Harris, 1963; Camalet et al., 2000; Boedecker et al., 2012; Huang and Doiron, 2017; Wilting and Priesemann, 2018a,b). Hence the principle of dynamic adaptive computation detailed below can be implemented and exploited in very diverse types of neural networks, and thus presents a general framework.

**Figure 2:**
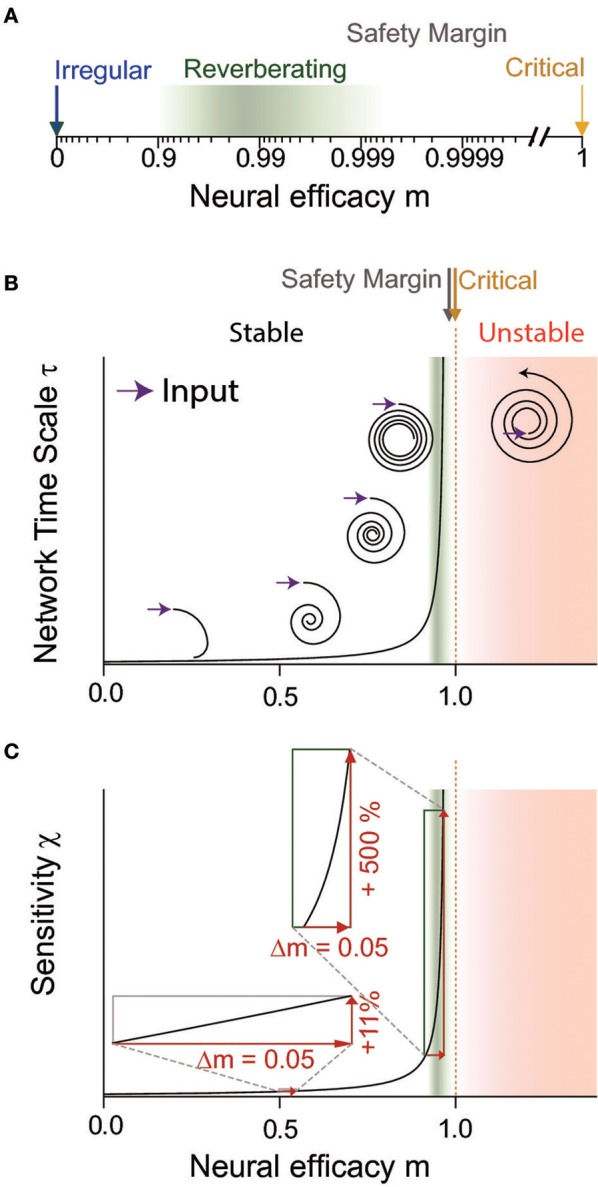
The neural efficacy *m* determines the average impact any spike has on the network. Depending on *m*, network dynamics can range from irregular (*m* = 0) to critical (*m* = 1) and unstable (*m* > 1) dynamics. **(A)** In a logarithmic depiction of *m*, the “reverberating regime” (green) observed for cortex *in vivo* is well visible. It has clearly a larger *m* than the irregular state (blue), but maintains a safety margin to criticality (yellow) and the instability associated with the supercritical regime (red in **B**,**C**). **(B,C)** Sketch to illustrate the divergence of dynamical and computational properties at a critical phase transition, at the example of the network timescale and the sensitivity, respectively. **(B)** The network timescale determines how long input is maintained in the network. While any rate change is rapidly quenched close to the irregular state (*m* = 0), input “reverberates” in the network activity for increasingly long timescales when approaching criticality (*m* = 1). In the reverberating regime, the network timescale is tens to hundreds of milliseconds. For *m* > 1, input is amplified by the network, implying instability (assuming a supercritical Hopf bifurcation here for illustration). The reverberating regime keeps a sufficient safety margin from this instability. **(C)** The reverberating regime found *in vivo* allows large tuning of the sensitivity by small changes of the neural efficacies (e.g., synaptic strength or excitation-inhibition balance), in contrast to states further away from criticality (insets).

The neural efficacy *m* is a statistical description of the effective recurrent activation, which takes into account both excitatory and inhibitory contributions. We here use it to focus on the mechanism of dynamic adaptive computation in an isolated setting, instead of including as many details as possible. We take this approach for two reasons. First, this abstraction allows to discuss possible generic principles for adaptation. Second, our approach enables us to assess the network state *m* and possible adaptation *m*(*t*) from experiments. The abstract adaptation principles we consider here can be implemented by numerous physiological mechanisms, including top-down attention, adaptation of synaptic strengths or neuronal excitability, dendritic processing, disinhibition, changes of the local gain, or up-and-down states (Hirsch and Gilbert, 1991; London and Häusser, 2005; Wilson, 2008; Piech et al., 2013; Ramalingam et al., 2013; Karnani et al., 2016).

Inferring the neural efficacy *m* experimentally is challenging, because only a tiny fraction of all neurons can be recorded with the required millisecond precision (Priesemann et al., 2009; Ribeiro et al., 2014; Levina and Priesemann, 2017). In fact, such spatial subsampling can lead to strong underestimation of correlations in networks, and subsequently of *m* (Wilting and Priesemann, 2018a,b). However, recently, a subsampling-invariant method has been developed that enables a precise quantification of *m* even from only tens of recorded neurons (Wilting and Priesemann, 2018a,b). Together with complementary approaches, either derived from the distribution of covariances or from a heuristic estimation, evidence is mounting that *m* is between ≈0.9 and ≈0.995, consistently for visual, somatosensory, motor and frontal cortices as well as hippocampus (Priesemann et al., 2014; Dahmen et al., 2016; Wilting and Priesemann, 2018a,b). Hence, collective spiking activity is neither fully asynchronous nor critical, but in a *reverberating regime* between the two, and any input persists for tens to hundreds of milliseconds (Murray et al., 2014; Priesemann et al., 2014; Hasson et al., 2015; Dahmen et al., 2016; Wilting and Priesemann, 2018a,b). In more detail, both Dahmen and colleagues as well as Wilting & Priesemann estimated the neural efficacy to be about *m* = 0.98, ranging from about 0.9 to 0.995 when assessing spiking activity in various cortical areas (Figures 1A, 2A). This magnitude of neural efficacy *m* implies intrinsic timescales of tens to hundreds of milliseconds. Such intrinsic timescales were directly estimated from cortical recordings in macaque by Murray and colleagues, who identified a hierarchical organization of timescales across somatosensory, medial temporal, prefrontal, orbitofrontal, and anterior cingulate cortex—indicating that every cortical area has adapted its response properties to its role in information processing (Murray et al., 2014). These findings are also in agreement with the experiments by London and colleagues, who directly probed the neural efficacy by stimulating a single neuron in barrel cortex. They found the response to last at least 50 ms in primary sensory cortex, implying *m* ≳ 0.92 (London et al., 2010; Wilting and Priesemann, 2018a,b).

We will show that this reverberating regime with median *m* = 0.98 (0.9 < *m* < 0.995) observed *in vivo* is optimal in preparing cortical networks for flexible adaptation to a given task, and meets all the requirements for *dynamic adaptive computation* we postulated above.

The reverberating regime allows tuning of computational properties by small, physiologically plausible changes of network parameters. This is because the closer a system is to criticality (*m* = 1), the more sensitive its properties are to small changes in the neural efficacy *m*, e.g., to any change of synaptic strength or excitation-inhibition ratio (Figure 2C). In the reverberating regime the network can draw on this sensitivity: inducing small overall synaptic changes allows to adapt network properties to task requirements over a wide range. Assuming for example an AR(1), Ornstein-Uhlenbeck, or branching process, increasing *m* from 0.94 to 0.99 leads to a six-fold increase in the sensitivity of the network (Figure 2C). Here, the sensitivity ∂*r*/∂*h* ~ (1−*m*)^−1^ describes how the average network rate *r* responds to changes of the input *h* (Wilting and Priesemann, 2018a). In contrast, the same absolute change from *m* = 0.5 to *m* = 0.55 only increases the sensitivity by about 11%. Similar relations apply to the network's amplification and intrinsic timescale (Wilting and Priesemann, 2018b). Thereby, any mechanism that increases or decreases the overall likelihood that a spike excites a postsynaptic neuron can mediate the change in neural efficacy *m*. Such mechanisms may act on the synaptic strength of many neurons in a given network, including neuromodulation that can change the response properties of a small population within a few hundred milliseconds, and homeostatic plasticity or long term potentiation or depression, which adapt the network over hours (Rang et al., 2003; Turrigiano and Nelson, 2004; Zierenberg et al., 2018). An alternative target could be the excitability of neurons, either rapidly by modulatory input, dendritic processing, disinhibition, changes of the local gain, or up-and-down states (Hirsch and Gilbert, 1991; London and Häusser, 2005; Wilson, 2008; Larkum, 2013; Piech et al., 2013; Ramalingam et al., 2013; Karnani et al., 2016); or by changes of the intrinsic conductance properties over hours to days (Turrigiano et al., 1994).

While states even closer to the phase transition than *m* = 0.98 would imply even stronger sensitivity to changes in *m*, being too close to the phase transition comes with the risk of crossing over to instability (*m* > 1), because synapses are altered continuously by a number of processes, ranging from depression and facilitation to long-term plasticity. Hence, posing a system too close to a critical phase transition may lead to instabilities and potentially causes epileptic seizures (Meisel et al., 2012; Priesemann et al., 2014; Wilting and Priesemann, 2018a). In the following, we estimate that the typical synaptic variability limits the precision of network tuning, and thereby defines an optimal regime for functional tuning that is about one percent away from the phase transition. Typically, single synapses exhibit about 50% variability in their strengths *w*, i.e., σ_*w*_ ≈ 0.5*w* over the course of hours and days (Statman et al., 2014). If these fluctuations are not strongly correlated across synapses, the variance of the single neuron efficacy $m_{i}\approx k\bar{w}$ scales with the number *k* of outgoing synapses, $\sigma_{{\;m}_{i}}^{2}\approx k\sigma_{w}^{2}$ and gives

(1) $$\begin{array}{l} \sigma_{m_{i}}\approx 0.5m_{i}/\sqrt{k}. \end{array}$$

Assuming the network keeps a “safety margin” from instability (*m* > 1) of three standard deviations renders the network stable 99.9% of the time. The remaining, transient excursions into the unstable regime may be tolerable, because even in a slightly supercritical regime (*m* ⪆ 1), runaway activity occurs only rarely (Harris, 1963; Zierenberg et al., 2018). Thus assuming on average k=O(10,000) synapses per neuron (DeFelipe et al., 2002) yields that a safety margin of about 1.5% from criticality is sufficient to establish stability. The safety margin can be even smaller if one assumes furthermore that the variability of *m*_*i*_ among neurons in a local network is not strongly correlated, because the stability of network dynamics is determined by the average neural efficacy $m=\bar{m_{i}}$, not by the individual efficacies. Furthermore, network structure might also contribute to stabilizing network activity (Kaiser and Simonotto, 2010). The resulting margin from criticality is compatible with the *m* observed *in vivo*.

The generic model reproduces statistical properties of networks where excitatory and inhibitory dynamics can be described by an effective excitation, e.g., because of a tight balance between excitation and inhibition (Sompolinsky et al., 1988; Vreeswijk and Sompolinsky, 1996; Ostojic, 2014; Kadmon and Sompolinsky, 2015; Huang and Doiron, 2017). In general, models should operate in a regime with *m* < 1 to maintain long-term stability and a safety-margin. However, transient and strong stimulus-induced activation is key for certain types of computation, such as direction selectivity, or sub- and supra-linear summation, e.g., in networks with non-saturating excitation and feedback inhibition (Douglas et al., 1995; Suarez et al., 1995; Murphy and Miller, 2009; Lim and Goldman, 2013; Hennequin et al., 2014, 2017; Rubin et al., 2015; Miller, 2016). These networks show transient instability (i.e., *m*(*t*) > 1) until inhibition stabilizes the activity. Whether such transient, large changes in *m*(*t*) on a millisecond scale should be considered a “state” is an open question. Nonetheless, experimentally a time resolved *m*(*t*) can be estimated with high temporal resolution, e.g., in experiments with a trial-based design. This measurement could then give insight into the state changes required for computation.

Besides the sensitivity, a number of other network properties also diverge or are maximized at the critical point and are hence equally tunable under dynamic adaptive computation. They include the spatial correlation length, amplification, active information storage, trial-to-trial variability, and the intrinsic network timescale (Harris, 1963; Sethna, 2006; Boedecker et al., 2012; Barnett et al., 2013; Wilting and Priesemann, 2018a). Some of these properties, which diverge at the critical point as (1−*m*)^−β^ (with a specific scaling exponent β), are advantageous for a given task; others, in contrast, may be detrimental. For example, at criticality the trial-to-trial variability diverges and undermines reliable responses. Moreover, in the vicinity of the critical point convergence to equilibrium slows down (Scheffer et al., 2012; Shriki and Yellin, 2016). Thus, network fine-tuning most likely is not based on optimizing one single network response property alone, but represents a trade-off between desirable and detrimental aspects. This tradeoff can be represented in the most simple case by a goal function

(2) $$\begin{array}{l} \Phi_{\alpha }=\Phi_{+}-\alpha \Phi_{-}\propto {\left(1-m\right)}^{\beta_{+}}-\alpha^{\prime }{\left(1-m\right)}^{\beta_{-}}, \end{array}$$

which weighs the desired (Φ_+_) and detrimental (Φ_−_) aspects by a task dependent weight factor α, and might be called free energy in the sense of Friston (2010). Close to a phase transition, the desired and detrimental aspects diverge and depend on the critical scaling exponents β_+_ and β_−_. In this case, the normalization constants of Φ_+_ and Φ_−_ are taken into account by the rescaled weight α′. Maximizing the goal function then yields an optimal neural efficacy m^*^, which is here given by

(3) $$\begin{array}{l} m^{*}=1-{\left(\frac{\alpha^{\prime }\beta_{-}}{\beta_{+}}\right)}^{-\frac{1}{\beta_{+}-\beta_{-}}}. \end{array}$$

This optimal neural efficacy (i) is in a subcritical regime unless α = 0 (i.e., detrimental aspects do not matter) and (ii) depends on the weight α′ and the exponents β_+_ and β_−_. In the simplified picture of branching processes, the resulting m^*^ determines a large set of response properties, which can thus only be varied simultaneously. An “ideal” network should combine the capability of dynamic adaptive computation with the ability to tune many response properties independently. To which extent such a network is conceivable at all and how it would have to be designed is an open question.

One particularly important network property is the intrinsic network timescale τ. In many processes this intrinsic network timescale emerges from recurrent activation and is connected to the neural efficacy as τ = −Δ*t*/log(*m*) ≈ Δ*t*/(1 − *m*) (Wilting and Priesemann, 2018a), where Δ*t* is a typical lag of spike propagation from the presynaptic to the postsynaptic neuron. Input reverberates in the network over this timescale τ, and can thereby enable short-term memory without any changes in synaptic strength (Figure 2B). It has been proposed before that cortical computation relies on reverberating activity (Buonomano and Merzenich, 1995; Herz and Hopfield, 1995; Wang, 2002). Reverberations are also at the core of reservoir computing in echo state networks and liquid state machines (Maass et al., 2002; Jaeger et al., 2007; Boedecker et al., 2012). Here, we extend on this concept and propose that cortical networks not only rely on reverberations, but specifically harness the reverberating regime in order to change their computational properties, in particular the specific τ, amplification, and sensitivity depending on needs.

We expect dynamic adaptive computation to fine-tune computational properties when switching from one task to the next, potentially mediated by neuromodulators, but we also expect that with development every brain area or circuit has developed computational properties that match their respective role in processing. Experimentally, evidence for a developmental or evolutionary tuning has been provided by Murray and colleagues, who showed that cortical areas developed a hierarchical organization as detailed above, with somatosensory areas showing fast responses (τ ≈ 100 ms), and frontal slower ones (τ ≈ 300 ms) (Murray et al., 2014). This hierarchy indicates that the ground-state dynamics of cortical circuits is indeed precisely tuned, and it is hypothesized that the hierarchical organization provides increasingly larger windows for information integration for example across the visual hierarchy (Hasson et al., 2008, 2015; Badre and D'Esposito, 2009; Chaudhuri et al., 2015; Chen et al., 2015). In addition to that backbone of hierarchical cortical organization, dynamic adaptive computation enables the fine-tuning of a given local circuit to specific task conditions. Indeed, experimental studies have shown that the response properties of cortical networks clearly change with task condition and cognitive state (Kisley and Gerstein, 1999; Massimini et al., 2005; Poulet and Petersen, 2008; Curto et al., 2009; Goard and Dan, 2009; Kohn et al., 2009; Harris and Thiele, 2011; Marguet and Harris, 2011; Priesemann et al., 2013; Scholvinck et al., 2015). Relating these changes to specific functional task requirements remains a theoretical and experimental challenge for the future.

## Author contributions

All authors were involved in the conception and revision of this study. JW, JZ, and VP wrote the manuscript. JW, JZ, and VP calculated the presented derivations. JW, JP, and VP drafted the figures.

### Conflict of interest statement

The authors declare that the research was conducted in the absence of any commercial or financial relationships that could be construed as a potential conflict of interest.
